# Supplementary material for: Gain or Loss? Evidence for Legume Predisposition to Symbiotic Interactions with Rhizobia via Loss of Pathogen-Resistance-Related Gene Families
Source: Int J Mol Sci. 2022 Dec 15;23(24):16003. doi: 10.3390/ijms232416003 (PMC9783688; doi:10.3390/ijms232416003)
Supplement: Supplementary file 1 [file ijms-23-16003-s001.zip › Supplementary Figures S1-S19- Annotated phylogenetic trees.pdf]

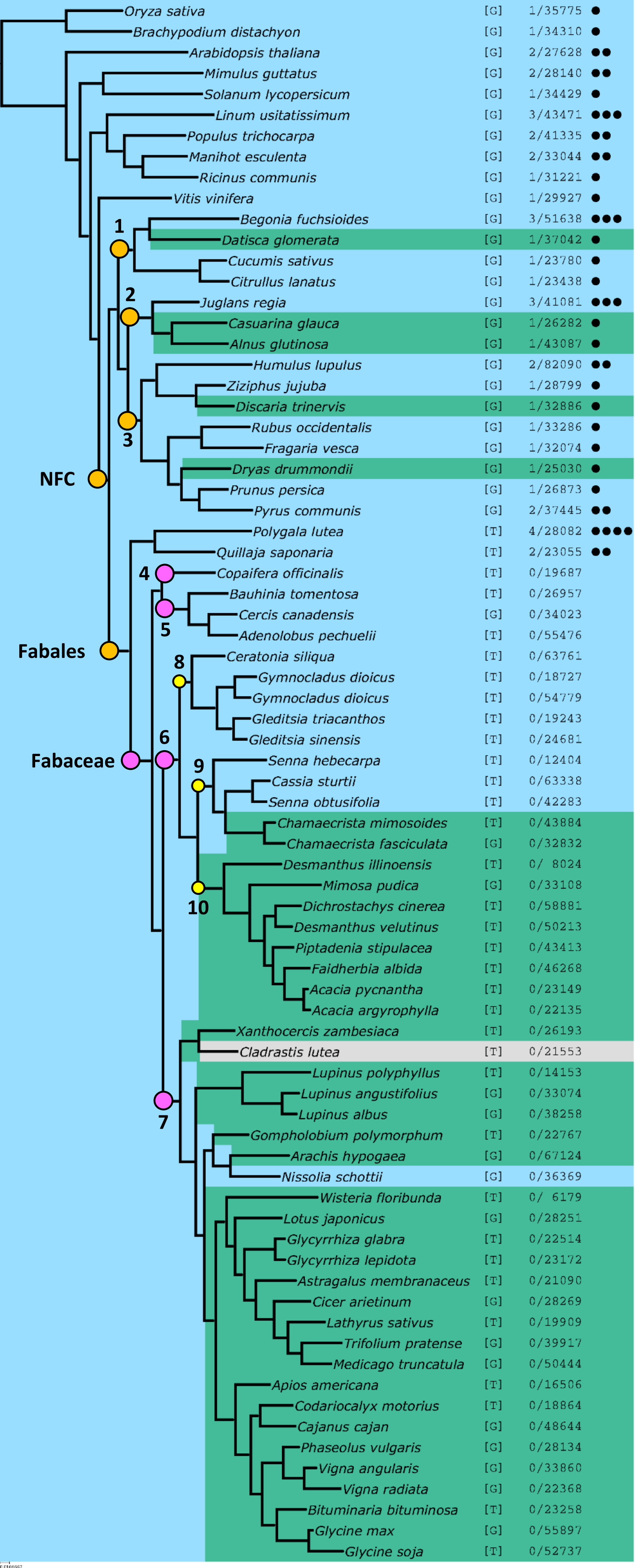

Supplementary Figure S1: Phylogenetic tree depicting the evolutionary relationships of 74 angiosperm plant species and distribution of orthologous genes assigned to orthogroup OG0011803 (Hapless protein). Orthologue frequency is visually indicated by black dots and numerically presented as a proportion of the total gene content observed in the species transcriptome [T] or genome [G]. Nodes of important lineages are highlighted (and numbered): orange for the nitrogen fixing clade (NFC) and its four formative orders (Fabales; 1, Cucurbitales; 2, Fagales; and 3, Rosales); pink for the Fabaceae family and four of its six subfamilies (4, Detarioideae; 5, Cercidoideae; 6, Caesalpinioideae; and 7, Papilionoideae); and yellow for three Caesalpinioideae clades (8, *Umtiza*; 9, *Cassia*; and 10, Mimosoid). The capacity of each species to form root nodules for symbiotic nitrogen fixation is represented by blue (non-nodulating), green (nodulating) and grey (undetermined) shading.

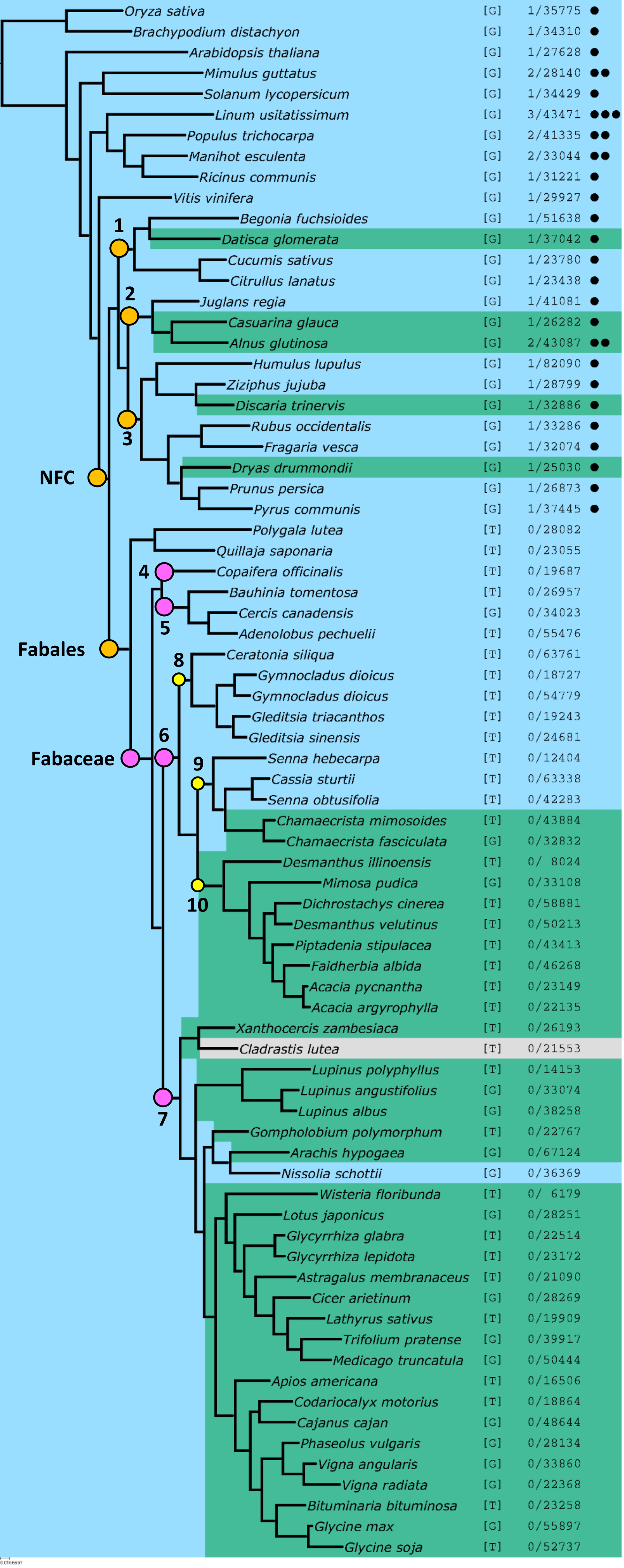

Supplementary Figure S2: Phylogenetic tree depicting the evolutionary relationships of 74 angiosperm plant species and distribution of orthologous genes assigned to orthogroup OG0012406 (Aspartyl protease family protein 2). Orthologue frequency is visually indicated by black dots and numerically presented as a proportion of the total gene content observed in the species transcriptome [T] or genome [G]. Nodes of important lineages are highlighted (and numbered): orange for the nitrogen fixing clade (NFC) and its four formative orders (Fabales; 1, Cucurbitales; 2, Fagales; and 3, Rosales); pink for the Fabaceae family and four of its six subfamilies (4, Detarioideae; 5, Cercidoieae; 6, Caesalpinioideae; and 7, Papilionoideae); and yellow for three Caesalpinioideae clades (8, *Umtiza*; 9, *Cassia*; and 10, Mimosoid). The capacity of each species to form root nodules for symbiotic nitrogen fixation is represented by blue (non-nodulating), green (nodulating) and grey (undetermined) shading.

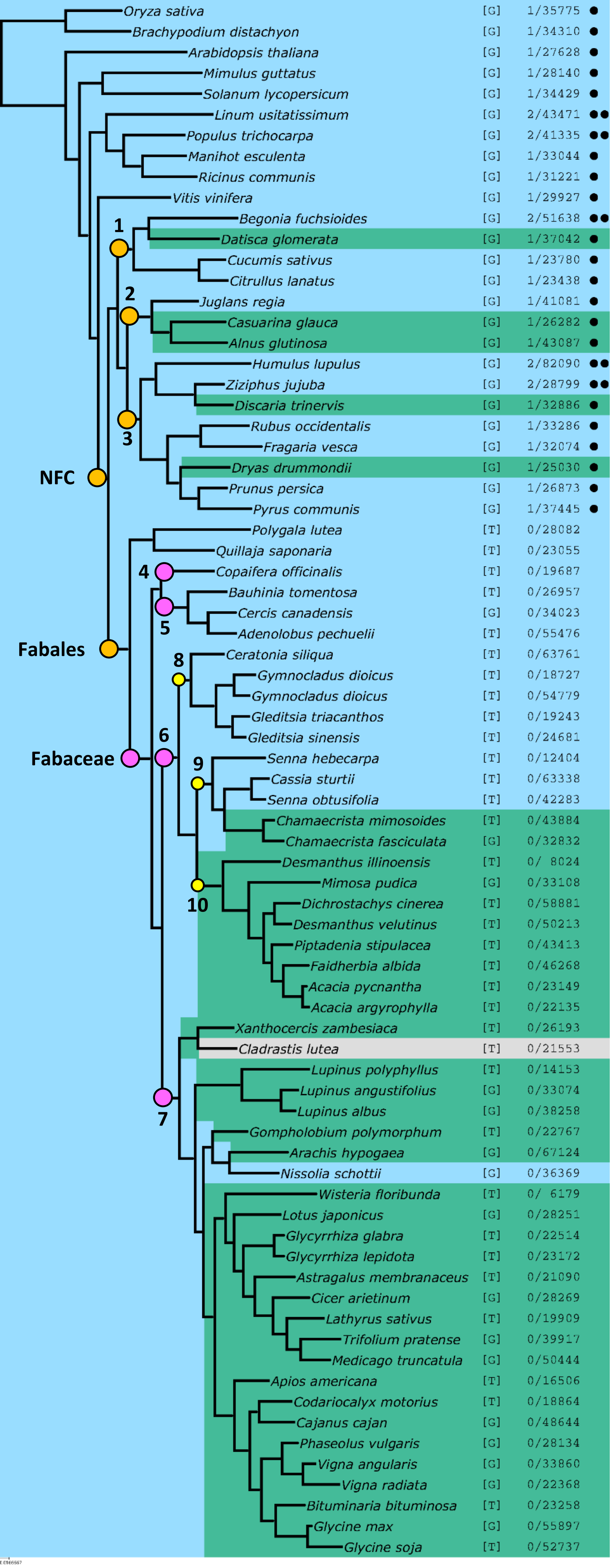

Supplementary Figure S3: Phylogenetic tree depicting the evolutionary relationships of 74 angiosperm plant species and distribution of orthologous genes assigned to orthogroup OG0012477 (Arabinogalactan protein 20). Orthologue frequency is visually indicated by black dots and numerically presented as a proportion of the total gene content observed in the species transcriptome [T] or genome [G]. Nodes of important lineages are highlighted (and numbered): orange for the nitrogen fixing clade (NFC) and its four formative orders (Fabales; 1, Cucurbitales; 2, Fagales; and 3, Rosales); pink for the Fabaceae family and four of its six subfamilies (4, Detarioideae; 5, Cercidoideae; 6, Caesalpinioideae; and 7, Papilionoideae); and yellow for three Caesalpinioideae clades (8, *Umtiza*; 9, *Cassia*; and 10, Mimosoid). The capacity of each species to form root nodules for symbiotic nitrogen fixation is represented by blue (non-nodulating), green (nodulating) and grey (undetermined) shading.

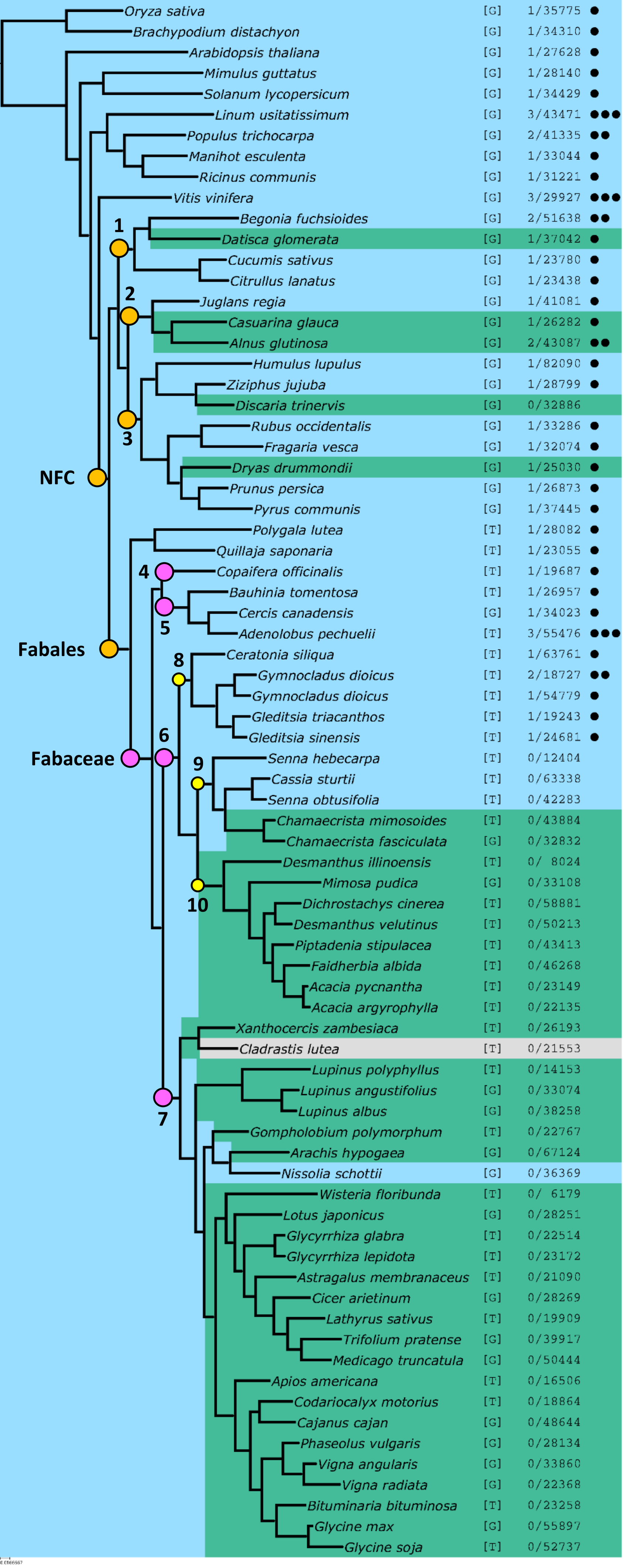

Supplementary Figure S4: Phylogenetic tree depicting the evolutionary relationships of 74 angiosperm plant species and distribution of orthologous genes assigned to orthogroup OG0011714 (Plant UBX domain-containing protein 11). Orthologue frequency is visually indicated by black dots and numerically presented as a proportion of the total gene content observed in the species transcriptome [T] or genome [G]. Nodes of important lineages are highlighted (and numbered): orange for the nitrogen fixing clade (NFC) and its four formative orders (Fabales; 1, Cucurbitales; 2, Fagales; and 3, Rosales); pink for the Fabaceae family and four of its six subfamilies (4, Detarioideae; 5, Cercidoideae; 6, Caesalpinioideae; and 7, Papilionoideae); and yellow for three Caesalpinioideae clades (8, *Umtiza*; 9, *Cassia*; and 10, Mimosoid). The capacity of each species to form root nodules for symbiotic nitrogen fixation is represented by blue (non-nodulating), green (nodulating) and grey (undetermined) shading.

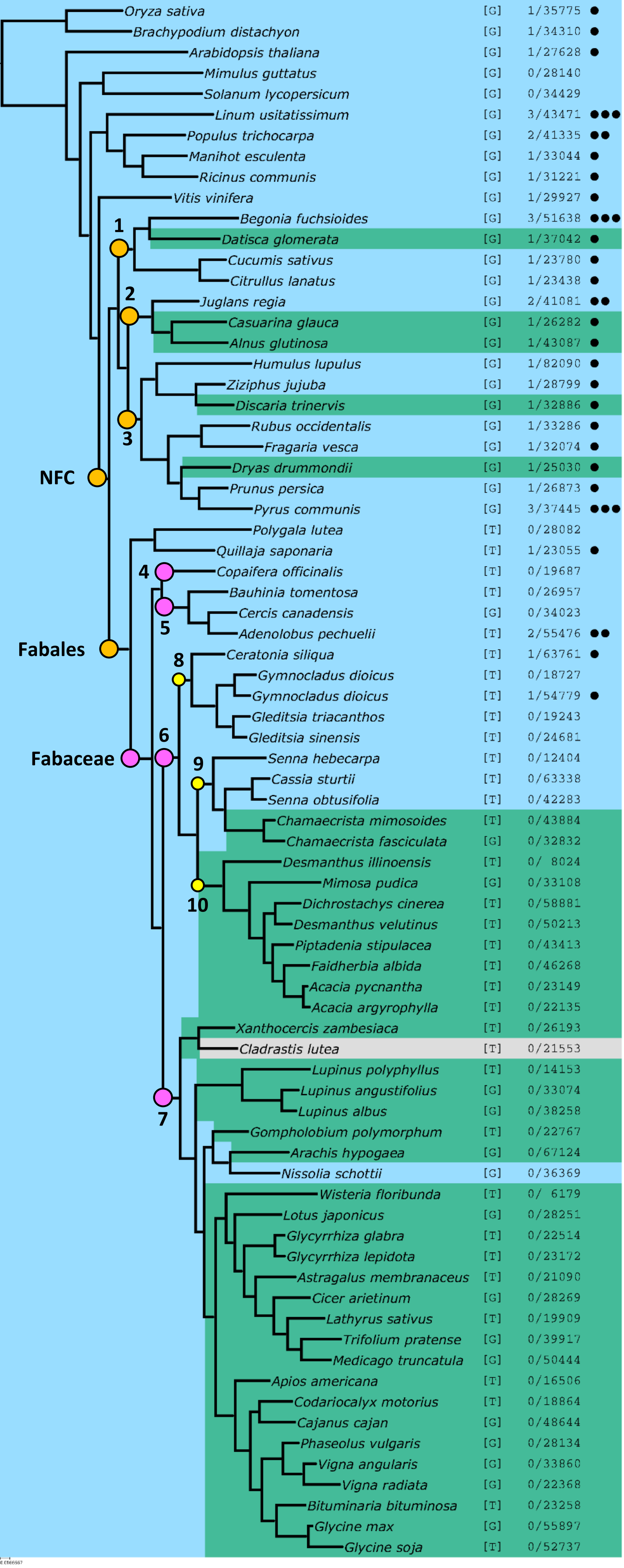

Supplementary Figure S5: Phylogenetic tree depicting the evolutionary relationships of 74 angiosperm plant species and distribution of orthologous genes assigned to orthogroup OG0012127 (Branchless trichome protein). Orthologue frequency is visually indicated by black dots and numerically presented as a proportion of the total gene content observed in the species transcriptome [T] or genome [G]. Nodes of important lineages are highlighted (and numbered): orange for the nitrogen fixing clade (NFC) and its four formative orders (Fabales; 1, Cucurbitales; 2, Fagales; and 3, Rosales); pink for the Fabaceae family and four of its six subfamilies (4, Detarioideae; 5, Cercidoieae; 6, Caesalpinioideae; and 7, Papilionoideae); and yellow for three Caesalpinioideae clades (8, *Umtiza*; 9, *Cassia*; and 10, Mimosoid). The capacity of each species to form root nodules for symbiotic nitrogen fixation is represented by blue (non-nodulating), green (nodulating) and grey (undetermined) shading.

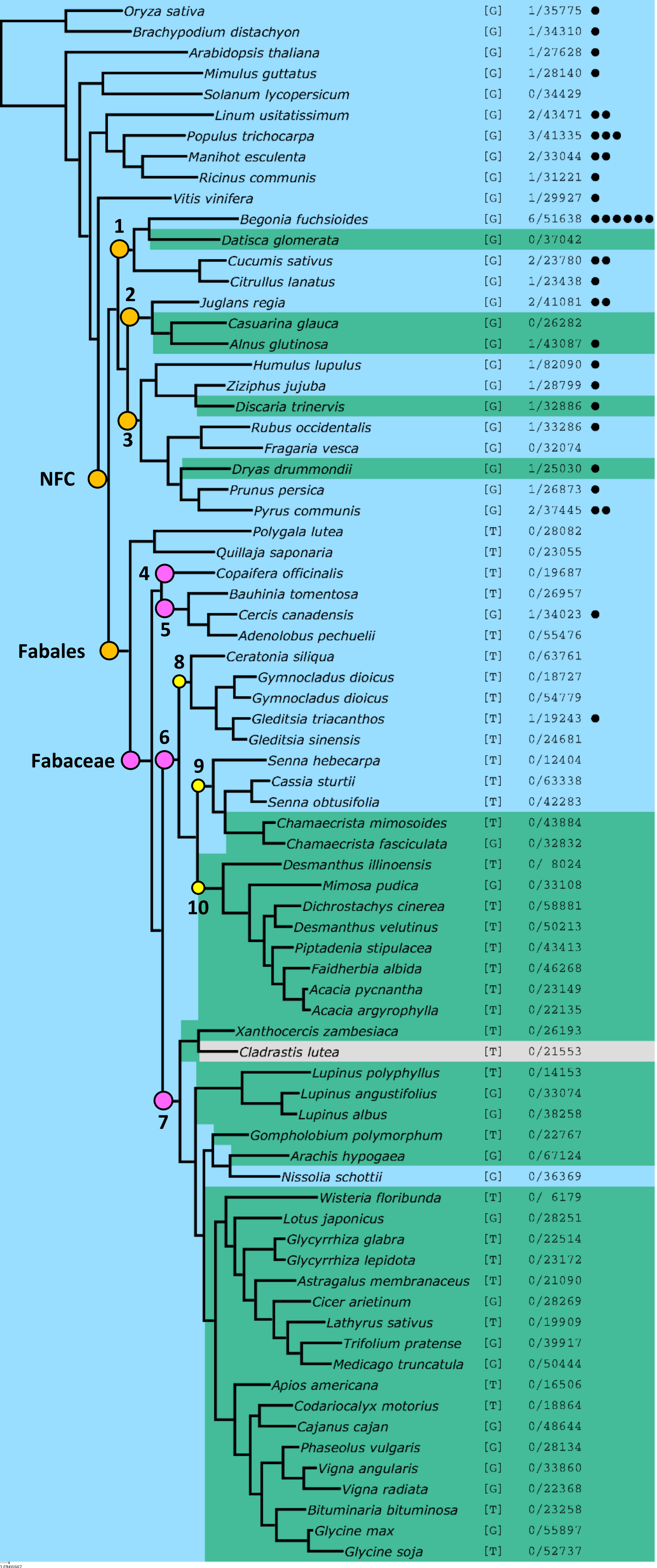

Supplementary Figure S6: Phylogenetic tree depicting the evolutionary relationships of 74 angiosperm plant species and distribution of orthologous genes assigned to orthogroup OG0012169 (TIFY, Jasmonate ZIM domain-containing protein). Orthologue frequency is visually indicated by black dots and numerically presented as a proportion of the total gene content observed in the species transcriptome [T] or genome [G]. Nodes of important lineages are highlighted (and numbered): orange for the nitrogen fixing clade (NFC) and its four formative orders (Fabales; 1, Cucurbitales; 2, Fagales; and 3, Rosales); pink for the Fabaceae family and four of its six subfamilies (4, Detarioideae; 5, Cercidoideae; 6, Caesalpinioideae; and 7, Papilionoideae); and yellow for three Caesalpinioideae clades (8, *Umtiza*; 9, *Cassia*; and 10, Mimosoid). The capacity of each species to form root nodules for symbiotic nitrogen fixation is represented by blue (non-nodulating), green (nodulating) and grey (undetermined) shading.

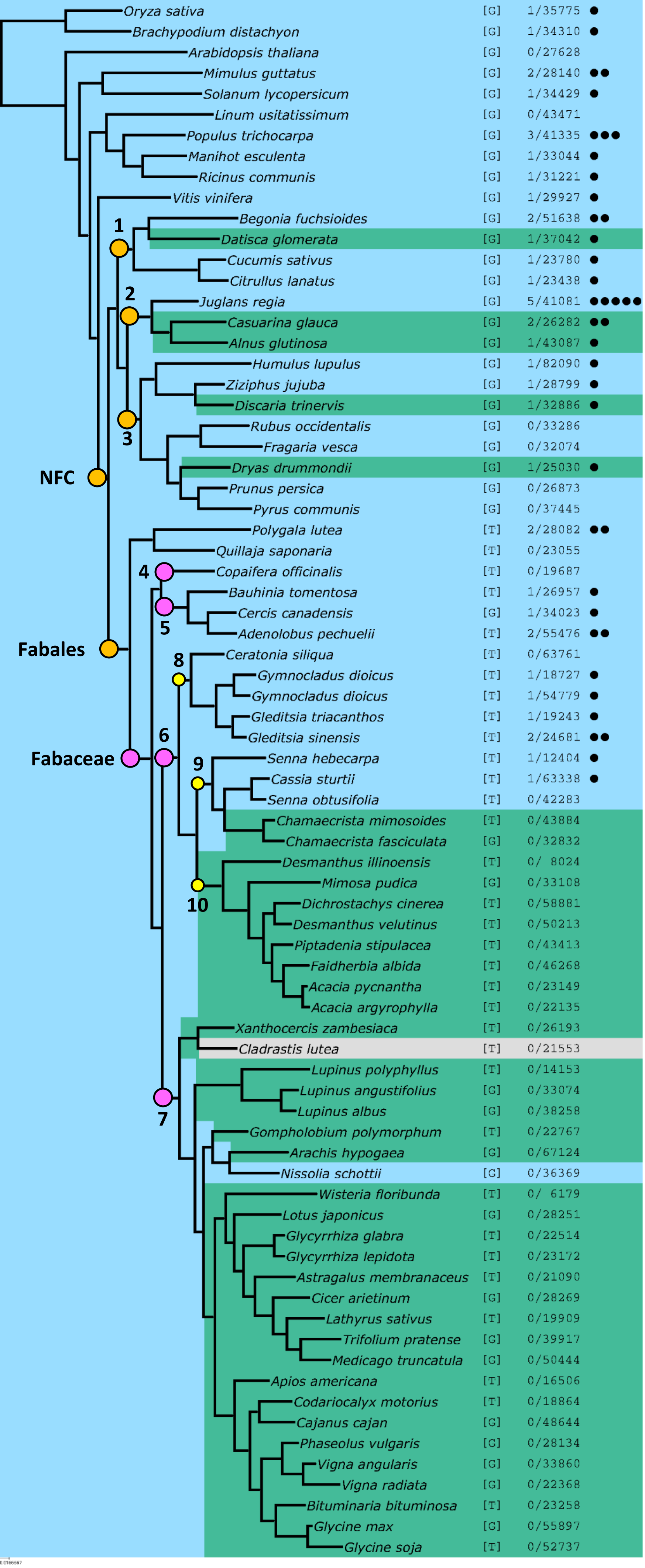

Supplementary Figure S7: Phylogenetic tree depicting the evolutionary relationships of 74 angiosperm plant species and distribution of orthologous genes assigned to orthogroup OG0011900 (F-box protein GID2). Orthologue frequency is visually indicated by black dots and numerically presented as a proportion of the total gene content observed in the species transcriptome [T] or genome [G]. Nodes of important lineages are highlighted (and numbered): orange for the nitrogen fixing clade (NFC) and its four formative orders (Fabales; 1, Cucurbitales; 2, Fagales; and 3, Rosales); pink for the Fabaceae family and four of its six subfamilies (4, Detarioideae; 5, Cercidoideae; 6, Caesalpinioideae; and 7, Papilionoideae); and yellow for three Caesalpinioideae clades (8, *Umtiza*; 9, *Cassia*; and 10, Mimosoid). The capacity of each species to form root nodules for symbiotic nitrogen fixation is represented by blue (non-nodulating), green (nodulating) and grey (undetermined) shading.

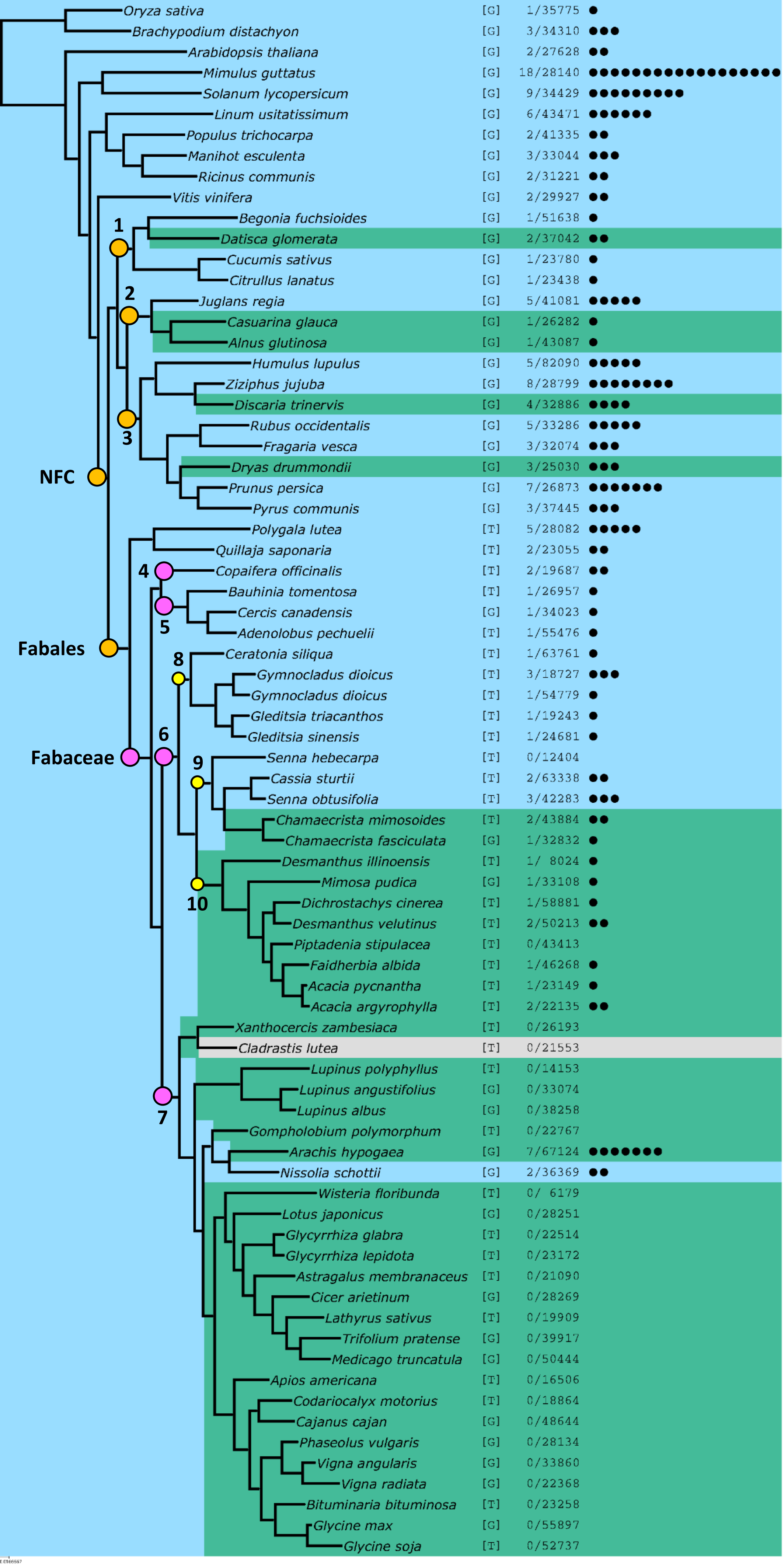

Supplementary Figure S8: Phylogenetic tree depicting the evolutionary relationships of 74 angiosperm plant species and distribution of orthologous genes assigned to orthogroup OG0004107 (UDP-glucosyltransferase 86A2). Orthologue frequency is visually indicated by black dots and numerically presented as a proportion of the total gene content observed in the species transcriptome [T] or genome [G]. Nodes of important lineages are highlighted (and numbered): orange for the nitrogen fixing clade (NFC) and its four formative orders (Fabales; 1, Cucurbitales; 2, Fagales; and 3, Rosales); pink for the Fabaceae family and four of its six subfamilies (4, Detarioideae; 5, Cercidoideae; 6, Caesalpinioideae; and 7, Papilionoideae); and yellow for three Caesalpinioideae clades (8, *Umtiza*; 9, *Cassia*; and 10, Mimosoid). The capacity of each species to form root nodules for symbiotic nitrogen fixation is represented by blue (non-nodulating), green (nodulating) and grey (undetermined) shading.

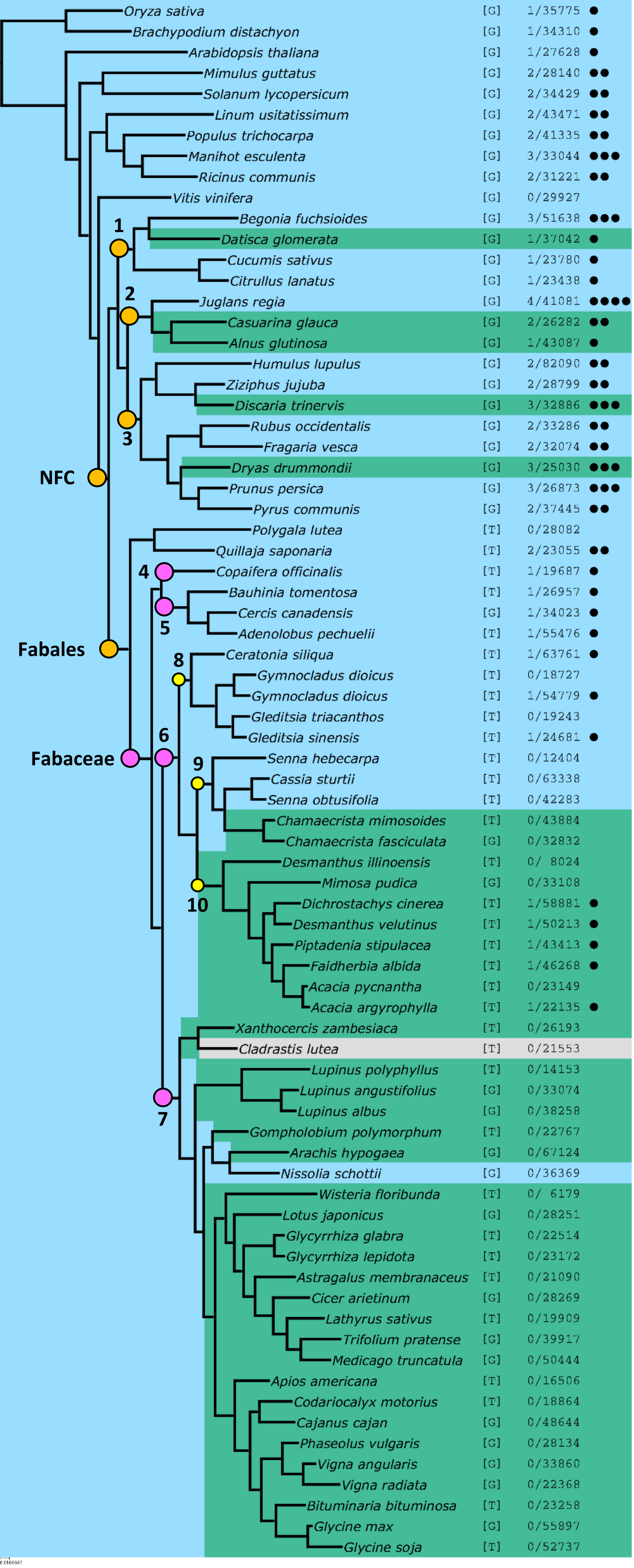

Supplementary Figure S9: Phylogenetic tree depicting the evolutionary relationships of 74 angiosperm plant species and distribution of orthologous genes assigned to orthogroup OG0010936 (Transcription factor IBH1). Orthologue frequency is visually indicated by black dots and numerically presented as a proportion of the total gene content observed in the species transcriptome [T] or genome [G]. Nodes of important lineages are highlighted (and numbered): orange for the nitrogen fixing clade (NFC) and its four formative orders (Fabales; 1, Cucurbitales; 2, Fagales; and 3, Rosales); pink for the Fabaceae family and four of its six subfamilies (4, Detarioideae; 5, Cercidoideae; 6, Caesalpinioideae; and 7, Papilionoideae); and yellow for three Caesalpinioideae clades (8, *Umtiza*; 9, *Cassia*; and 10, Mimosoid). The capacity of each species to form root nodules for symbiotic nitrogen fixation is represented by blue (non-nodulating), green (nodulating) and grey (undetermined) shading.

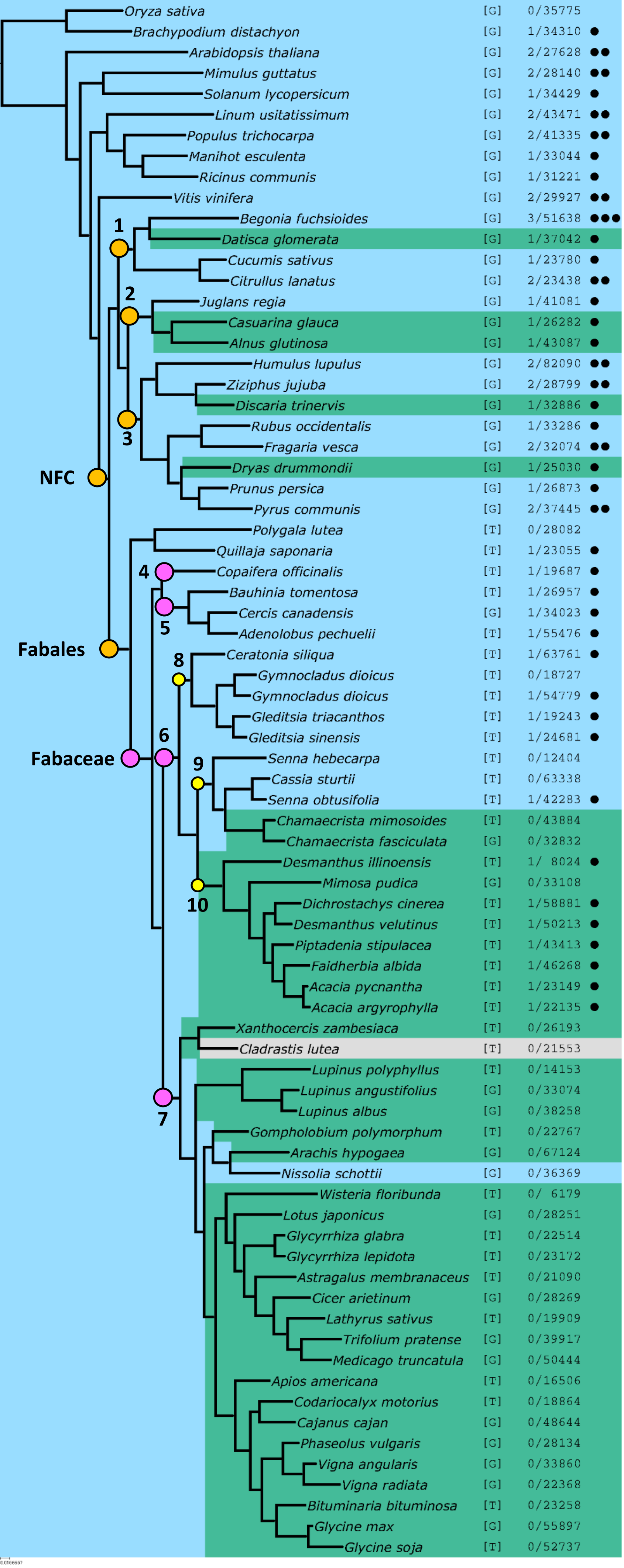

Supplementary Figure S10: Phylogenetic tree depicting the evolutionary relationships of 74 angiosperm plant species and distribution of orthologous genes assigned to orthogroup OG0011359 (Transducin/WD40 repeat-like superfamily protein). Orthologue frequency is visually indicated by black dots and numerically presented as a proportion of the total gene content observed in the species transcriptome [T] or genome [G]. Nodes of important lineages are highlighted (and numbered): orange for the nitrogen fixing clade (NFC) and its four formative orders (Fabales; 1, Cucurbitales; 2, Fagales; and 3, Rosales); pink for the Fabaceae family and four of its six subfamilies (4, Detarioideae; 5, Cercidoideae; 6, Caesalpinioideae; and 7, Papilionoideae); and yellow for three Caesalpinioideae clades (8, *Umtiza*; 9, *Cassia*; and 10, Mimosoid). The capacity of each species to form root nodules for symbiotic nitrogen fixation is represented by blue (non-nodulating), green (nodulating) and grey (undetermined) shading.

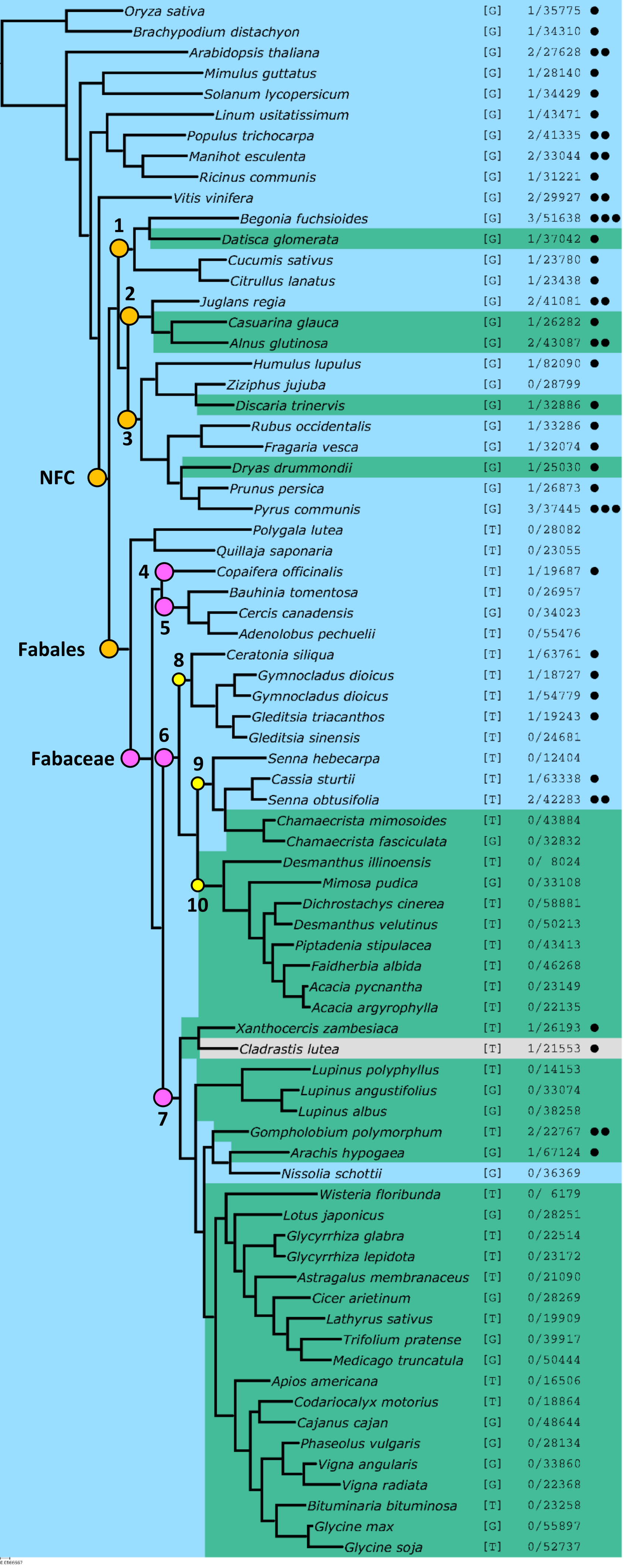

Supplementary Figure S11: Phylogenetic tree depicting the evolutionary relationships of 74 angiosperm plant species and distribution of orthologous genes assigned to orthogroup OG0011634 (unknown). Orthologue frequency is visually indicated by black dots and numerically presented as a proportion of the total gene content observed in the species transcriptome [T] or genome [G]. Nodes of important lineages are highlighted (and numbered): orange for the nitrogen fixing clade (NFC) and its four formative orders (Fabales; 1, Cucurbitales; 2, Fagales; and 3, Rosales); pink for the Fabaceae family and four of its six subfamilies (4, Detarioideae; 5, Cercidoieae; 6, Caesalpinioideae; and 7, Papilionoideae); and yellow for three Caesalpinioideae clades (8, *Umtiza*; 9, *Cassia*; and 10, Mimosoid). The capacity of each species to form root nodules for symbiotic nitrogen fixation is represented by blue (non-nodulating), green (nodulating) and grey (undetermined) shading.

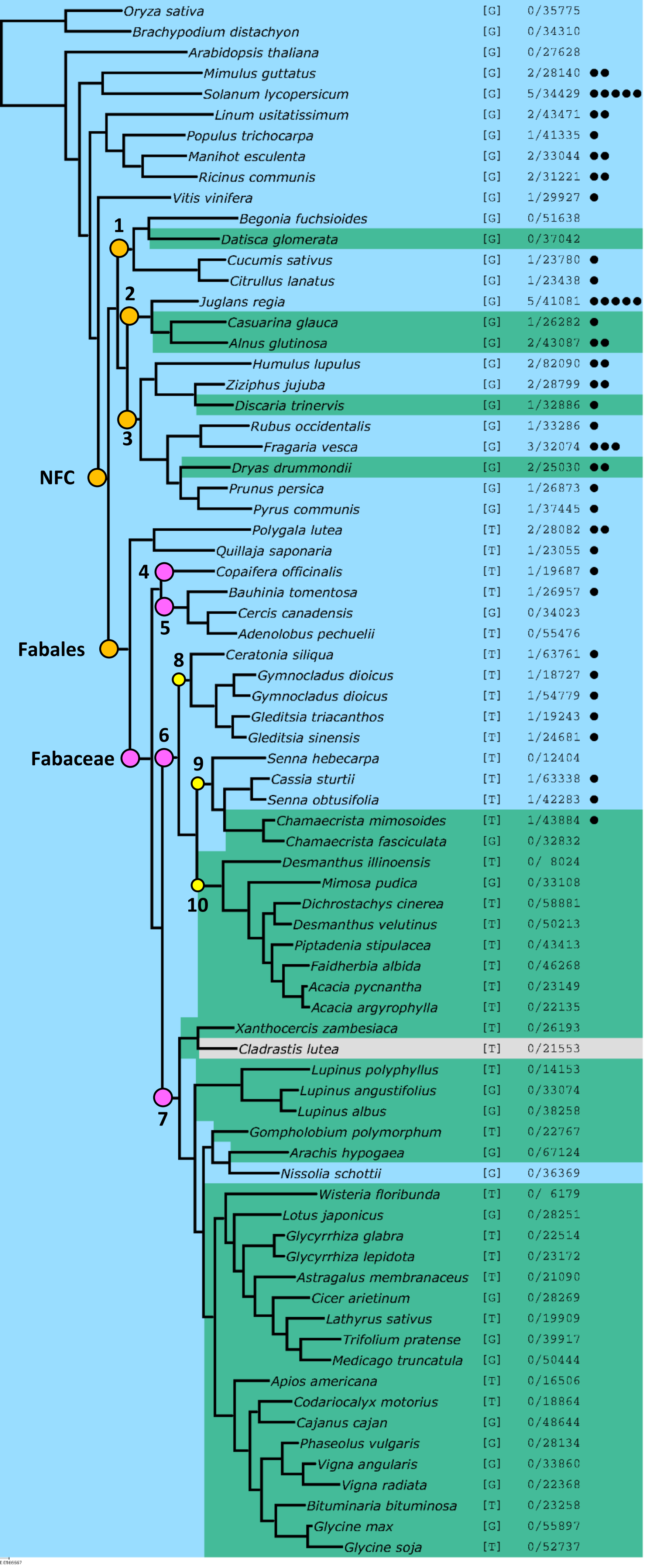

Supplementary Figure S12: Phylogenetic tree depicting the evolutionary relationships of 74 angiosperm plant species and distribution of orthologous genes assigned to orthogroup OG0011465 (F-box protein CPR1-like). Orthologue frequency is visually indicated by black dots and numerically presented as a proportion of the total gene content observed in the species transcriptome [T] or genome [G]. Nodes of important lineages are highlighted (and numbered): orange for the nitrogen fixing clade (NFC) and its four formative orders (Fabales; 1, Cucurbitales; 2, Fagales; and 3, Rosales); pink for the Fabaceae family and four of its six subfamilies (4, Detarioideae; 5, Cercidoieae; 6, Caesalpinioideae; and 7, Papilionoideae); and yellow for three Caesalpinioideae clades (8, *Umtiza*; 9, *Cassia*; and 10, Mimosoid). The capacity of each species to form root nodules for symbiotic nitrogen fixation is represented by blue (non-nodulating), green (nodulating) and grey (undetermined) shading.

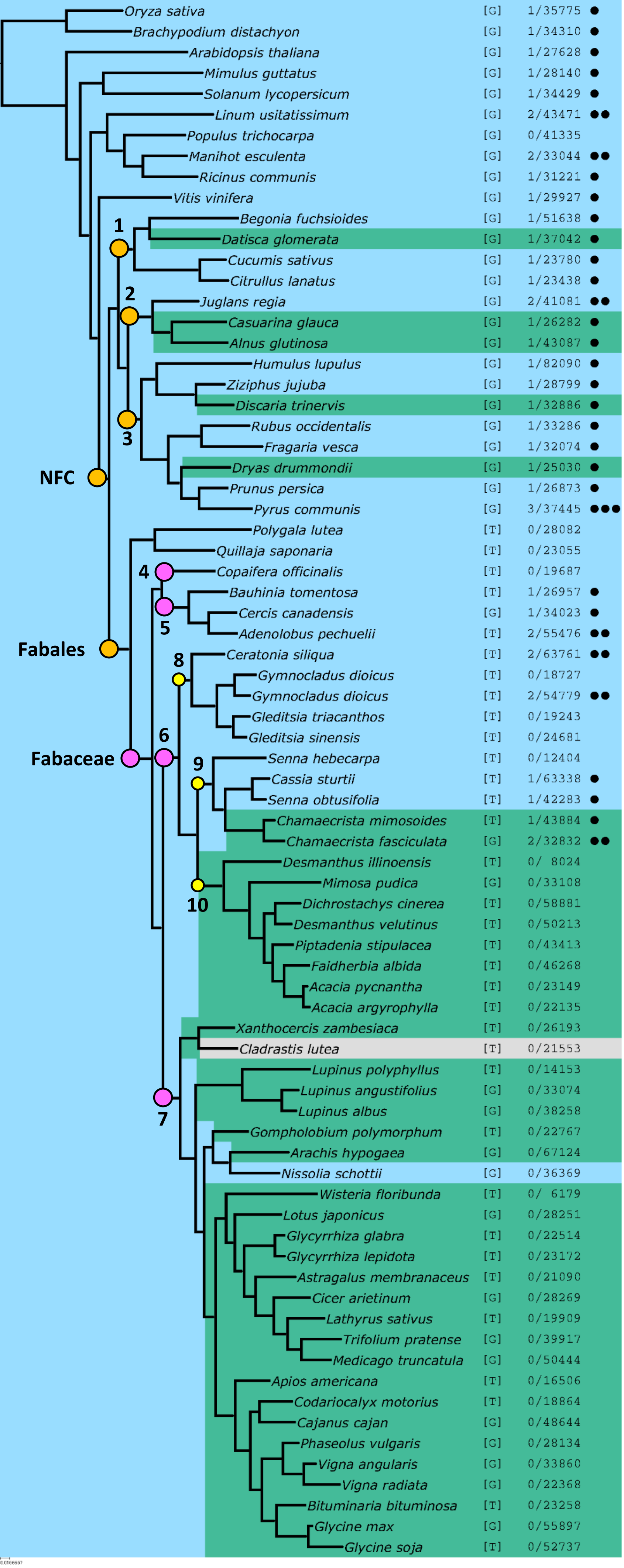

Supplementary Figure S13: Phylogenetic tree depicting the evolutionary relationships of 74 angiosperm plant species and distribution of orthologous genes assigned to orthogroup OG0011851 (Kinesin-like protein KIN-8B). Orthologue frequency is visually indicated by black dots and numerically presented as a proportion of the total gene content observed in the species transcriptome [T] or genome [G]. Nodes of important lineages are highlighted (and numbered): orange for the nitrogen fixing clade (NFC) and its four formative orders (Fabales; 1, Cucurbitales; 2, Fagales; and 3, Rosales); pink for the Fabaceae family and four of its six subfamilies (4, Detarioideae; 5, Cercidoideae; 6, Caesalpinioideae; and 7, Papilionoideae); and yellow for three Caesalpinioideae clades (8, *Umtiza*; 9, *Cassia*; and 10, Mimosoid). The capacity of each species to form root nodules for symbiotic nitrogen fixation is represented by blue (non-nodulating), green (nodulating) and grey (undetermined) shading.

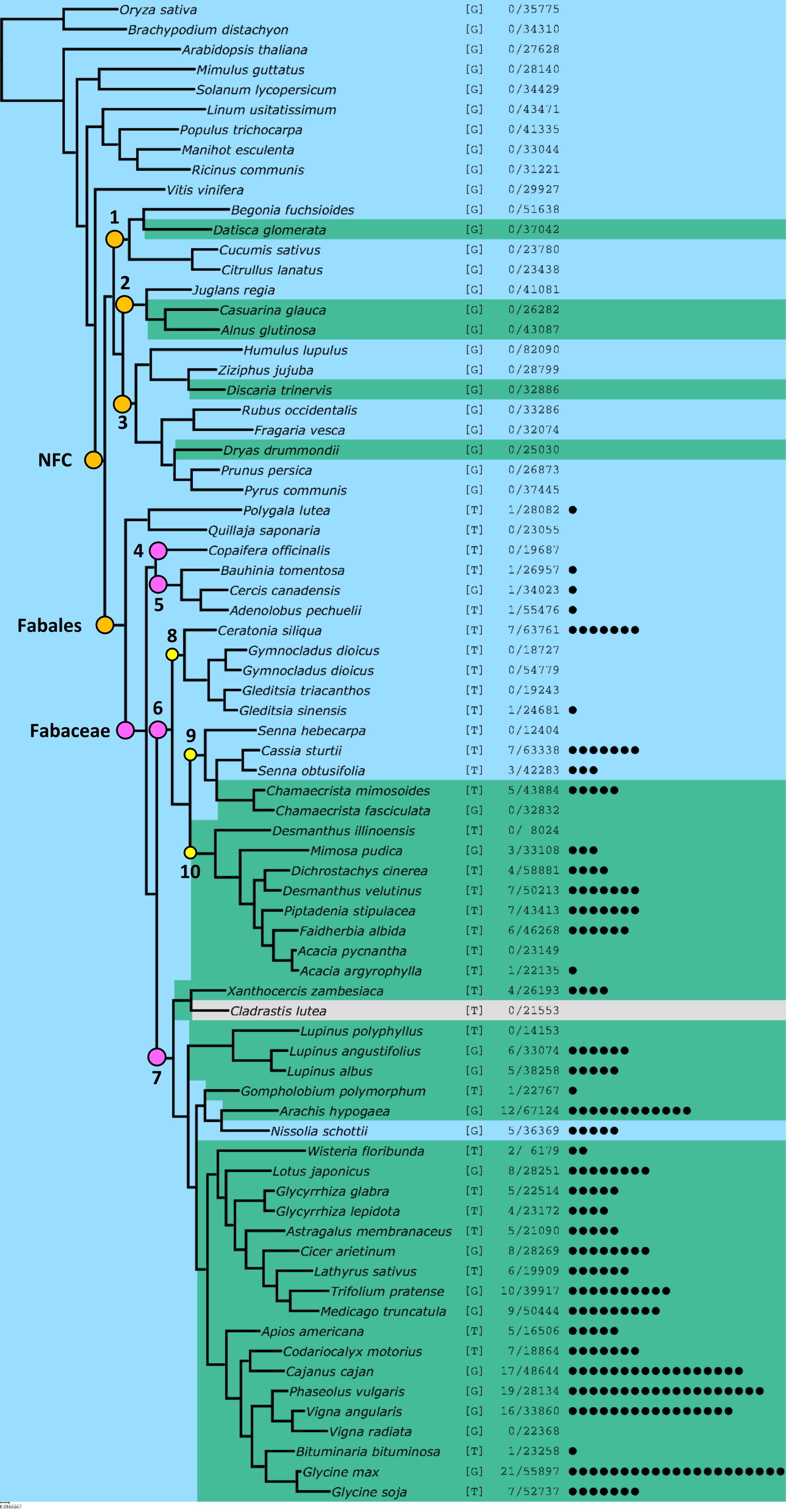

Supplementary Figure S14: Phylogenetic tree depicting the evolutionary relationships of 74 angiosperm plant species and distribution of orthologous genes assigned to orthogroup OG0001813 (Fasciclin-like arabinogalactan protein 11). Orthologue frequency is visually indicated by black dots and numerically presented as a proportion of the total gene content observed in the species transcriptome [T] or genome [G]. Nodes of important lineages are highlighted (and numbered): orange for the nitrogen fixing clade (NFC) and its four formative orders (Fabales; 1, Cucurbitales; 2, Fagales; and 3, Rosales); pink for the Fabaceae family and four of its six subfamilies (4, Detarioideae; 5, Cercidoideae; 6, Caesalpinioideae; and 7, Papilionoideae); and yellow for three Caesalpinioideae clades (8, *Umtiza*; 9, *Cassia*; and 10, Mimosoid). The capacity of each species to form root nodules for symbiotic nitrogen fixation is represented by blue (non-nodulating), green (nodulating) and grey (undetermined) shading.

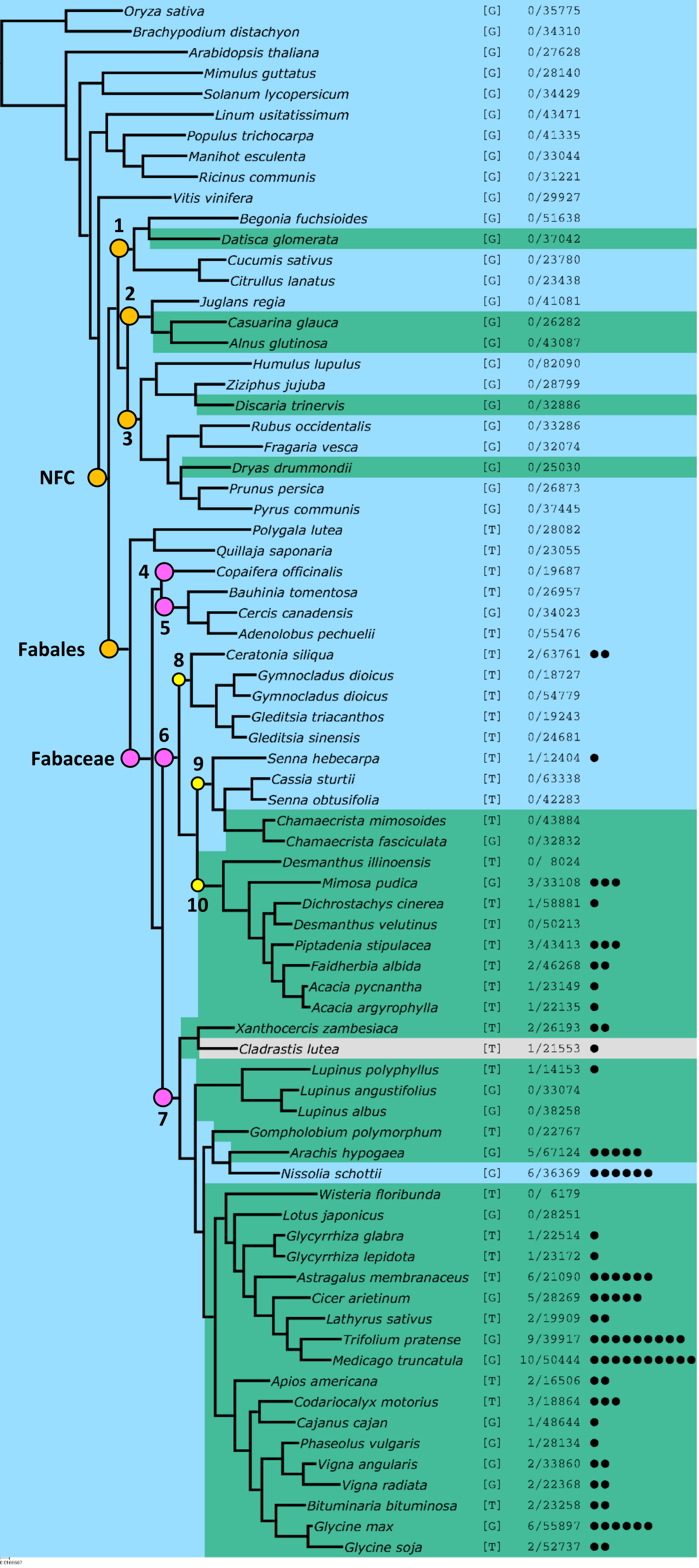

Supplementary Figure S15: Phylogenetic tree depicting the evolutionary relationships of 74 angiosperm plant species and distribution of orthologous genes assigned to orthogroup OG0009254 (Embryonic abundant protein USP92). Orthologue frequency is visually indicated by black dots and numerically presented as a proportion of the total gene content observed in the species transcriptome [T] or genome [G]. Nodes of important lineages are highlighted (and numbered): orange for the nitrogen fixing clade (NFC) and its four formative orders (Fabales; 1, Cucurbitales; 2, Fagales; and 3, Rosales); pink for the Fabaceae family and four of its six subfamilies (4, Detarioideae; 5, Cercidoideae; 6, Caesalpinioideae; and 7, Papilionoideae); and yellow for three Caesalpinioideae clades (8, *Umtiza*; 9, *Cassia*; and 10, Mimosoid). The capacity of each species to form root nodules for symbiotic nitrogen fixation is represented by blue (non-nodulating), green (nodulating) and grey (undetermined) shading.

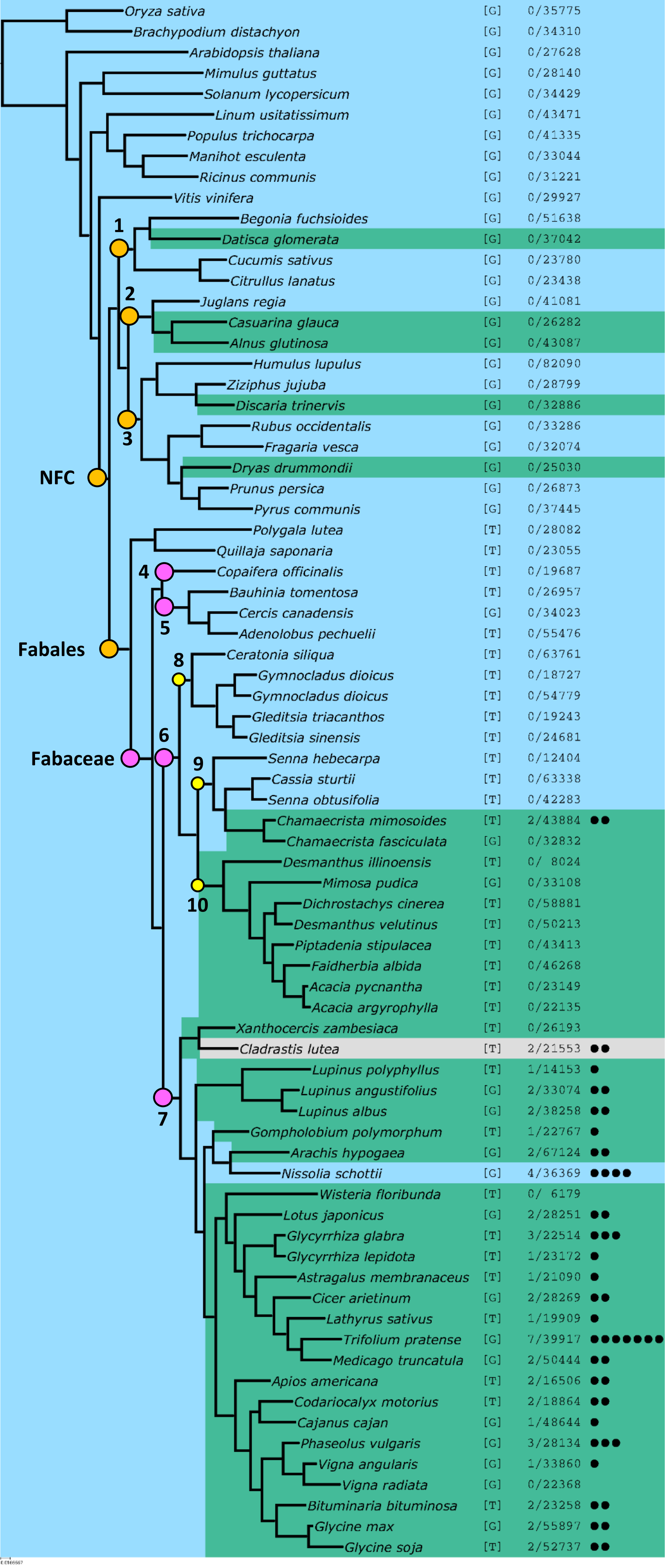

Supplementary Figure S16: Phylogenetic tree depicting the evolutionary relationships of 74 angiosperm plant species and distribution of orthologous genes assigned to orthogroup OG0011516 (Dirigent protein 21). Orthologue frequency is visually indicated by black dots and numerically presented as a proportion of the total gene content observed in the species transcriptome [T] or genome [G]. Nodes of important lineages are highlighted (and numbered): orange for the nitrogen fixing clade (NFC) and its four formative orders (Fabales; 1, Cucurbitales; 2, Fagales; and 3, Rosales); pink for the Fabaceae family and four of its six subfamilies (4, Detarioideae; 5, Cercidoideae; 6, Caesalpinioideae; and 7, Papilionoideae); and yellow for three Caesalpinioideae clades (8, *Umtiza*; 9, *Cassia*; and 10, Mimosoid). The capacity of each species to form root nodules for symbiotic nitrogen fixation is represented by blue (non-nodulating), green (nodulating) and grey (undetermined) shading.

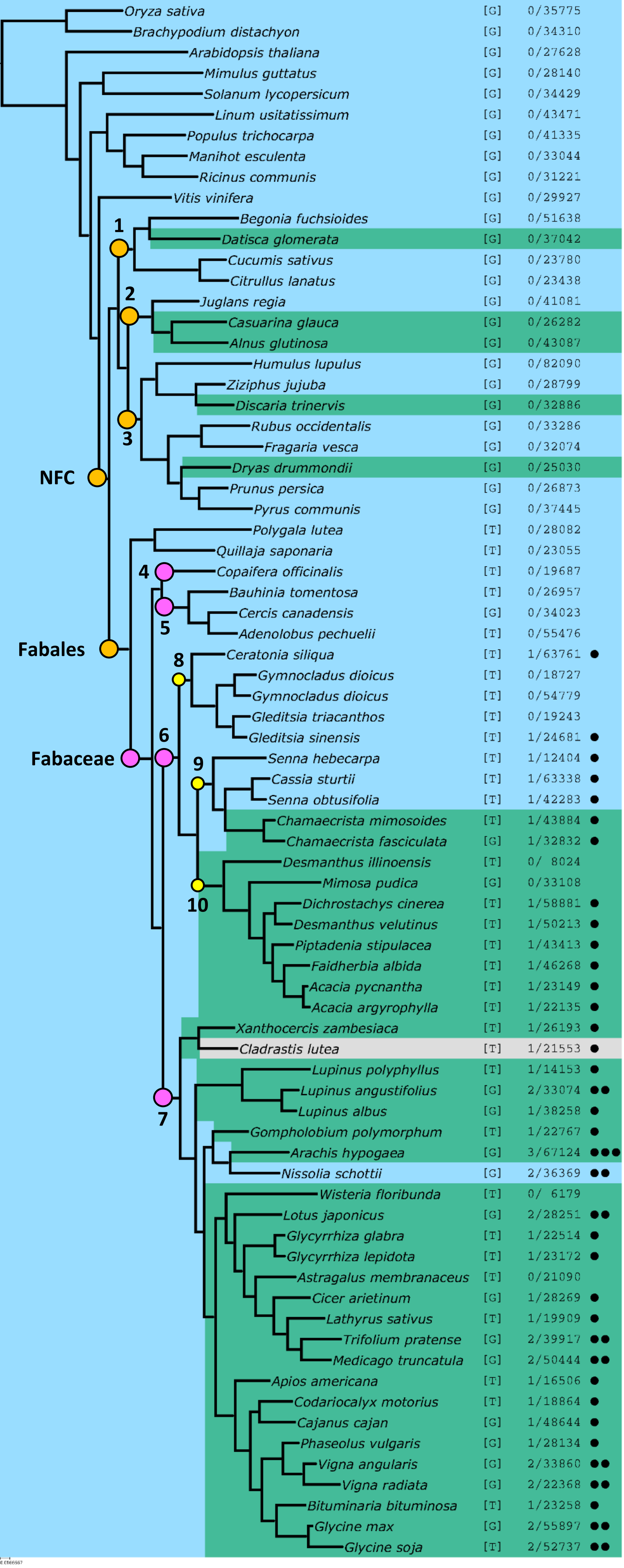

Supplementary Figure S17: Phylogenetic tree depicting the evolutionary relationships of 74 angiosperm plant species and distribution of orthologous genes assigned to orthogroup OG0011581 (60S acidic ribosomal protein P0). Orthologue frequency is visually indicated by black dots and numerically presented as a proportion of the total gene content observed in the species transcriptome [T] or genome [G]. Nodes of important lineages are highlighted (and numbered): orange for the nitrogen fixing clade (NFC) and its four formative orders (Fabales; 1, Cucurbitales; 2, Fagales; and 3, Rosales); pink for the Fabaceae family and four of its six subfamilies (4, Detarioideae; 5, Cercidoideae; 6, Caesalpinioideae; and 7, Papilionoideae); and yellow for three Caesalpinioideae clades (8, *Umtiza*; 9, *Cassia*; and 10, Mimosoid). The capacity of each species to form root nodules for symbiotic nitrogen fixation is represented by blue (non-nodulating), green (nodulating) and grey (undetermined) shading.

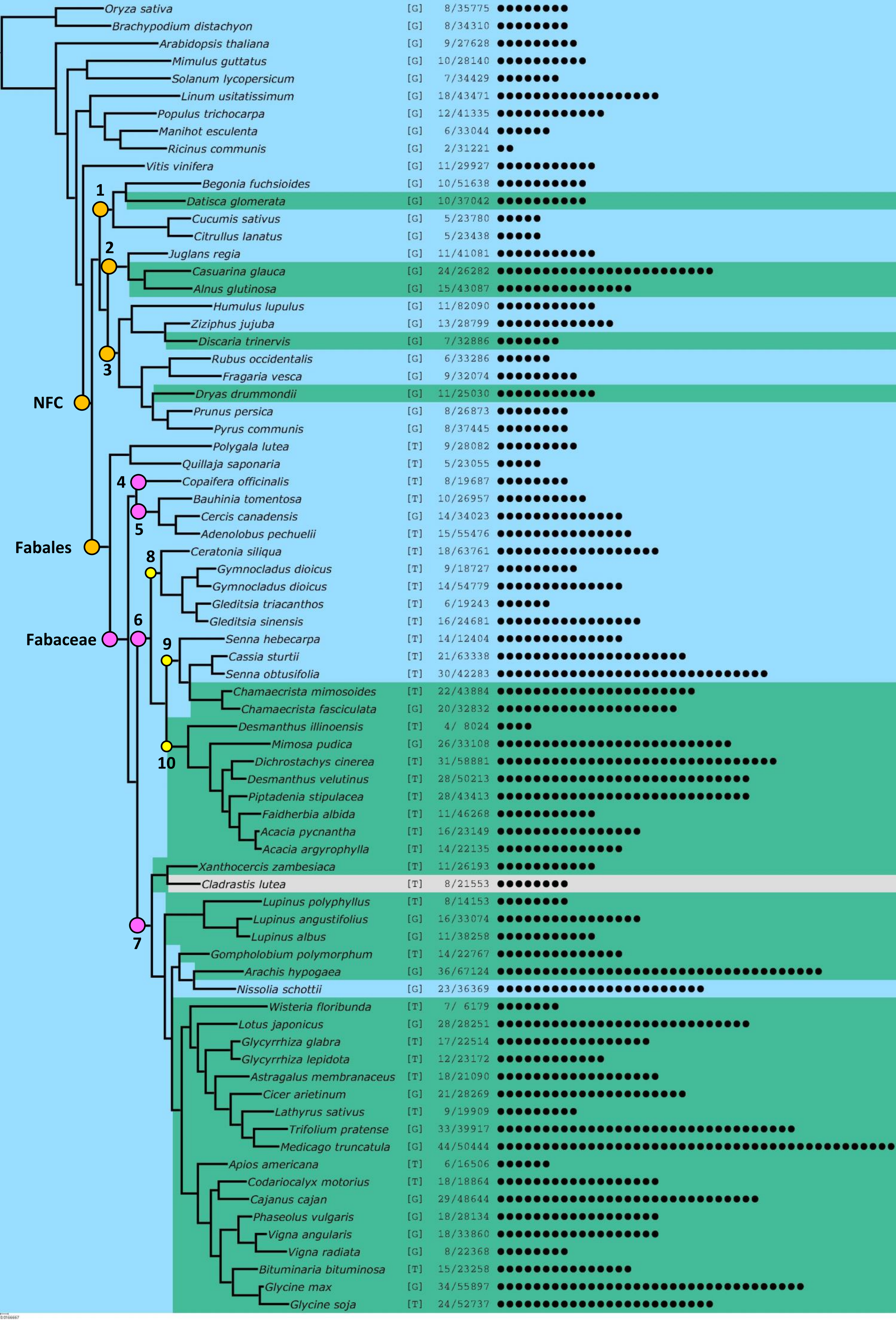

Supplementary Figure S18: Phylogenetic tree depicting the evolutionary relationships of 74 angiosperm plant species and distribution of orthologous genes assigned to orthogroup OG0000085 (UDP-glucosyltransferase protein). Orthologue frequency is visually indicated by black dots and numerically presented as a proportion of the total gene content observed in the species transcriptome [T] or genome [G]. Nodes of important lineages are highlighted (and numbered): orange for the nitrogen fixing clade (NFC) and its four formative orders (Fabales; 1, Cucurbitales; 2, Fagales; and 3, Rosales); pink for the Fabaceae family and four of its six subfamilies (4, Detarioideae; 5, Cercidoideae; 6, Caesalpinioideae; and 7, Papilionoideae); and yellow for three Caesalpinioideae clades (8, *Umtiza*; 9, *Cassia*; and 10, Mimosoid). The capacity of each species to form root nodules for symbiotic nitrogen fixation is represented by blue (non-nodulating), green (nodulating) and grey (undetermined) shading.

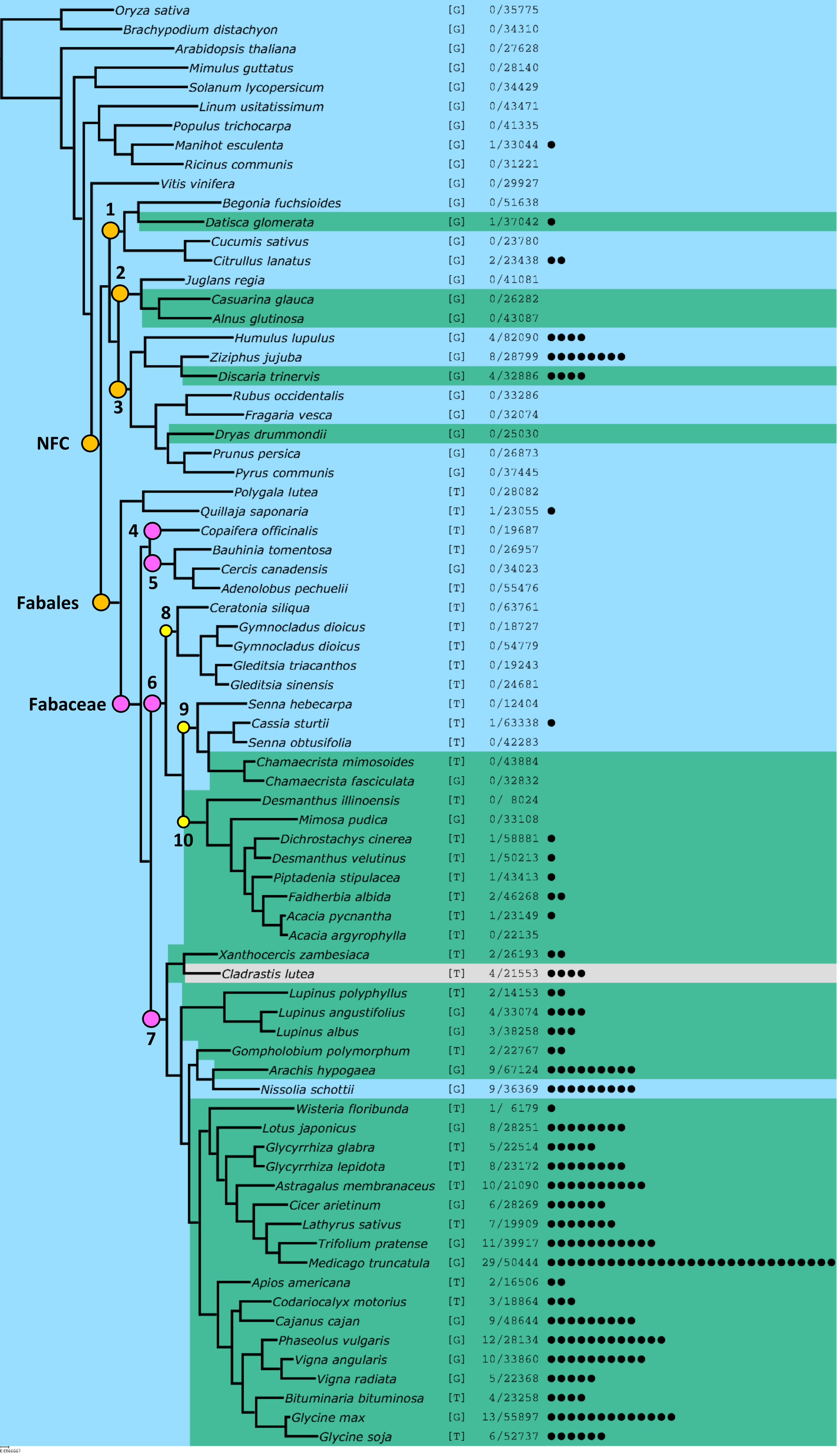

Supplementary Figure S19: Phylogenetic tree depicting the evolutionary relationships of 74 angiosperm plant species and distribution of orthologous genes assigned to orthogroup OG0002264 (Soyasapogenol B glucuronide galactosyltransferase). Orthologue frequency is visually indicated by black dots and numerically presented as a proportion of the total gene content observed in the species transcriptome [T] or genome [G]. Nodes of important lineages are highlighted (and numbered): orange for the nitrogen fixing clade (NFC) and its four formative orders (Fabales; 1, Cucurbitales; 2, Fagales; and 3, Rosales); pink for the Fabaceae family and four of its six subfamilies (4, Detarioideae; 5, Cercidoideae; 6, Caesalpinioideae; and 7, Papilionoideae); and yellow for three Caesalpinioideae clades (8, *Umtiza*; 9, *Cassia*; and 10, Mimosoid). The capacity of each species to form root nodules for symbiotic nitrogen fixation is represented by blue (non-nodulating), green (nodulating) and grey (undetermined) shading.
